# The Late Dr. John Armstrong

**Published:** 1830-01-01

**Authors:** 


### THE LATE DR. JOHN ARMSTRONG.

"Nothing extenuate, nor aught set down in malice."

---

On Saturday, the 12th December, 1829, MEDICAL SCIENCE lost one of its most zealous and talented cultivators, in the person of Dr. Armstrong—cut off in the prime of life, (46 years of age)—in the spring-tide of professional estimation and emolument—a successful practitioner, an applauded teacher, a generous rival, and a kind friend! That Dr. Armstrong had enemies, is not to be wondered at. Envy is—

"A shade that follows wealth and fame,"

and could no more fail to pursue Dr. Armstrong than his own shadow in full sunshine. In none of his printed works did he ever retort upon his enemies individually, or use a single harsh expression towards any of his cotemporaries—a fact that authorises the epithet (generous rival) which we have applied to him.

The first appearance of Dr. Armstrong's work on Typhus Fever, in 1816, led us to bestow a warm encomium on the merits of the book and the talents of its author—and, since that period, we have not ceased to do justice (we trust) to the abilities of a physician who was our competitor and contemporary—but to whom we never owed a single obligation beyond those arising out of common friendly intercourse. We have been accused, however, of over-rating the talents and lauding too highly the merits of Dr. Armstrong—and we may have done so—for, "to err is human;" but now that the subject of this notice is gone to eternal repose; when all living ties are dissolved, and all feelings of friendship, fear, envy, gratitude, or jealousy, (if they ever did exist,) are left to their free course, we have no hesitation in reiterating our first and our last sentiments respecting Dr. Armstrong when alive.

1817.

"To the author himself (Dr. A.) we beg leave to offer our sincere congratulations on his valuable performance. Excepting in the perusal and delineation of Dr. Parry's ELEMENTS of PATHOLOGY, we have not, for many years, derived so much pleasure in our analytical labours, as in the course of the present analysis. For the talents bestowed on Dr. Armstrong by Nature—for the experience gained by time—for the facts and observations treasured up by industry, he is accountable at the tribunal of humanity and the shrine of science —*Medico-Chirurgical Journal, Vol.* 3, *p.* 124, *Feb.* 1817.

1828.

"Dr. Armstrong's works on Typhus, &c. which appeared some ten or twelve years ago, procured for their author a reception and patronage from the professional and general public, such as have never before been witnessed or experienced in the annals of medicine. From being a successful author, Dr. A. became, in a very short time, one of the most successful candidates for private practice, in this great metropolis, whither he came a total stranger—and, from a successful career in practice, he turned his attention to medical tuition, where he quickly drew a larger class of students than any other physician in London. Some people may say this was all good fortune—or chance. But we have an idea, that to take such a lead in three different and difficult walks of the profession—writing, practising, and teaching—requires something more than the chapter of accidents, the patronage of friends, or the caprice of fashion."—*Med. Chir. Review. Vol.* IX. *p.* 347, *Aug.* 1828.

Thus, then, we have uniformly and steadfastly advocated the talents of Dr. Armstrong, from his first appearance on the stage (after the establishment of this Journal) up to the year before his death; and a review of our conduct does not induce us to alter our published opinions. As we said above, we may have *over-rated* Dr. Armstrong, but we do not think that our estimate of his talents was very false. In what way, we ask, can a physician's abilities be estimated, but by the general sense of his professional brethren? Now, Dr. Armstrong came to London in 1817 or 18, on the sole faith of the reception which his work on Typhus had experienced from the periodical press; and without any extra-professional patronage. In ten or twelve years he advanced to a private practice of four or five thousand pounds per annum, and he established the largest medical class in London, by lectures which were listened to by multitudes of applauding students, and reiterated in the periodicals of the day. His works have been re-published in America, and they have been quoted with approbation by authors of repute in every country. We say again, that all this could not have taken place, except by a very general sense of his merits diffused among the medical community at large.

Every one knows that the usual practice of mankind is to decry talent while living, and extol it to the skies when its possessor has paid the last debt of Nature.

> " All human virtue, to its latest breath,
> Finds envy never conquered but by death."

Not having followed the usual example of mankind in niggardly withholding approbation of the living, (when approbation is of any use)—we shall not imitate the other part of man's conduct, by heaping eulogies on the tomb, when the spirit is fled far beyond the reach of insult or adulation.

> " Can Honour's voice provoke the silent dust,
> Or Flattery soothe the dull cold ear of death?"

Many circumstances conspire to render it almost impossible for an exact estimate of a physician's public writings, or private character to be formed, or, at all events, proclaimed, during his life. The interests of science and of humanity, however, demand that TRUTH, divested of the panegyric of friends or detraction of enemies, should be established, when the individual has passed that bourn, beyond which the trumpet of FAME can never push its loudest note of good, or of evil report!

Having then, for a long time, bestowed our humble mead of approbation on the *published writings* of Dr. Armstrong, we shall now offer a few remarks on the *professional character* of the man himself;—remarks which, for obvious reasons, could never be made but in a post-obit biography. We trust that our past and present conduct will prove that we have acted up to the spirit of the motto at the head of this paper.

We venture to assert then, that the lamented physician, whose premature death has called forth this notice, was indebted to nature for splendid talents; and that he would have equally distinguished himself had he embraced any other department of literature, science, art, arms, or politics, instead of physic. That his original education, both classical and medical, was very scanty, he was neither ashamed nor afraid to declare to many of his friends, and on many occasions. His natural abilities, and his habitual industry soon rendered this primary defect a mere speck, that was scarcely discernible, and of no material consequence in the cultivation of medical science, where personal observation forms almost the whole circle of knowledge.

An acquaintance of eleven years with Dr Armstrong, induces us to aver that the distinguishing feature, the prominent character of his mind, and that to which he owed a great deal of his successful career, was an ARDENT AND MOST PROLIFIC IMAGINATION. While reading his works, or listening to his discourses, one would be tempted to consider him as entirely devoted to practical facts and observations, without any addiction to theory. Yet we have seldom known a more speculative physician engaged in actual practice. At the very commencement of his career in London, his mind appeared stored with, and he could pour forth in profusion, such a mass of observation and experience, in explanation of any phenomenon, or in illustration of any doctrine, as could hardly be collected by the most careful observer, during a long life of professional avocation. This resulted from an ardent and prolific imagination, multiplying deductions beyond all proportion to facts, and combining or arranging these deductions in the mind, till they appeared to the individual himself, and even to those around him, as a vast magazine of experience, which it would be unreasonable to doubt and difficult to oppose.

It was the same fertility of imagination that led to another remarkable trait in Dr. Armstrong's professional character, mutability of doctrine and opinion. Where deduction is exuberant in such a science as that of medicine, fallacy must be frequent; and the detection of error, by so acute and candid a mind as Dr. Armstrong's, would naturally lead to change of principles, and even of practice. Those, on the other hand, who are slow and cautious in drawing inferences; who allow facts to multiply and accumulate largely before they attempt to construct doctrines or principles, are not so liable to those fluctuations of opinion that lessen our confidence in the judgment, though they enhance our admiration of the candour of those who proclaim them.

To the very same propensity towards exuberant deduction from fertility of imagination, is to be attributed the eloquence of Dr. A. in his lectures, and the persuasive force of his arguments in conversation. It was quite impossible to bring him into any difficulties by force of argument. His resources were inexhaustible, and he overpowered his opponent with hosts of facts and observations, which could not be parried or paralleled.

The vacillations of doctrine, so conspicuous in Dr. Armstrong's writings and lectures, necessarily led to mutations of practice in the treatment of diseases. A stranger who had just perused his admirable work on typhus fever, where blood-letting, calomel and opium, purgation, &c. are so boldly prescribed, would have been astonished to see Dr. Armstrong a few years after the publication of his work, confining his measures to the exhibition of cold-drawn castor oil. Those who were best acquainted with Dr. Armstrong's private practice, can testify that he became not merely a cautious, but, in many respects, a timid practitioner.

The public declaration which this talented and ingenuous physician lately made, of his change of creed in respect to contagion, caused a strong sensation in medical society; and many of his best and ablest friends came to the conclusion that, on this point at least, a widening circle of experience tended rather to bewilder than clear the judgment. Dr. Armstrong began to see that this mutability of doctrine would prove, sooner or later, destructive of professional confidence in his practice; and, for the last two or three years of his life, he indulged less freely in the expression of his opinions, at least, in conversation.

Such were the chief, perhaps, the only defects or failings in the professional character of Dr. Armstrong. They were amply compensated by talents of the highest order, and dispositions of the most amiable nature. His industry, we think, has never had a parallel, and there cannot be a doubt that his mental and corporeal exertions, in preparing and delivering lectures, while carrying on an extensive practice, contributed much to undermine his health, and accelerate his death.

The career of success which the subject of this notice experienced during twelve years' residence in the metropolis, has never been equalled, as far as we are acquainted with the annals of medical men, in this or in any other country. His success, we have some reason to believe, has been nearly the ruin of several of his brethren, who were tempted to bring their talents into the great mart of London, on the strength of Dr. Armstrong's rapid advance in fame and fortune—but whose prospects were blighted by withering neglect, and ended in total failure. It is very well known that Dr. Armstrong came to London from Sunderland (in the beginning of 1818) with little or no patronage, and without having acquired more than a very moderate share of professional reputation in the scene of his first practice. His work on Fever, however, was in the course of a very flattering reception, and a vacancy in the London Fever Hospital, seemed to open a fine field for talent, and point at once to Dr. Armstrong as the person best qualified to cultivate that field with honour to himself and advantage to the public. Here one of those events which often determine a man's good or bad fortune through life, occurred, and seemed to threaten the very existence of his professional character! The canvas having commenced, Dr. A. repaired to the College of Physicians, and presented himself for examination and a license. He was rejected! The crisis of his fate was at hand—and he must have been gifted with second sight who could augur any thing but utter ruin from the astounding decision in Warwick-lane! The event of this apparent disaster affords a memorable example of the good which not unfrequently springs from evil. Nothing but a strong reaction in the public mind, against the fiat of the College and in favour of the rejected candidate for a license, could have saved Dr. Armstrong from total ruin. That reaction instantly took place. He was elected physician to the institution, with the full knowledge of his rejection by the censors appointed to test his medical knowledge—and a great many of the general practitioners in London, to their honour, took Dr. Armstrong's part, and materially promoted his introduction into private practice!

The mysterious rejection at the College has never been cleared up. That the censors, who were all men of honour, conceived that Dr. Armstrong was unfit to practise in London as a physician, until he had acquired more medical knowledge — or more knowledge of Greek and Latin, there can be no doubt—but the event falsified their decision—for not one of their learned body, whether fellow or licentiate, has, since that period, acquired half as much fame or emolument, as the rejected Armstrong!

Considering how often and how long the most splendid talents, the most extensive acquirements, the most unwearied attention, and the most honourable conduct, are destined to pine in the shade of this modern Babylon, there cannot be much doubt that the apparently sinister event just alluded to, was the basis, or, at all events, a most powerful auxiliary to the brilliant but short career which marked the path of him who now lies tranquil in the tomb! That his genius and industry —his mild manners—his amiable dispositions—and his zeal in the cause of humanity, science, and truth, would ultimately have surmounted every obstacle opposed to the rise of merit even in such times as these—and especially in such a metropolis as London, we believe —because, to all the foregoing qualities and qualifications, Dr. Armstrong added consummate TACT, and worldly wisdom, so that no man ever turned the favours of Fortune or the fruits of labour to a better account than he did—and that in the modest but attractive garb of simplicity and humility. Still we may safely conclude that, had it not been for the reaction in Doctor Armstrong's favour, occasioned by his rejection at the College, his professional path would have been rougher, and his progress slower than it was.

Be this as it may, it was not till within these seven or eight years, that his practice extended beyond the sum of two thousand pounds per annum. With this ratio of advancement, he was not satisfied, and he turned his ardent and active mind to the construction of a course of lectures, which are now before the public, and the success of which is well known. The celebrity of the man—the matter of the discourses—the animated and eloquent manner in which they were delivered—and the practice of elucidating diseases by numerous drawings and preparations, soon drew together a class of students far more numerous than that of any other medical teacher in this metropolis.

Dr. Armstrong's penetration had early led him to perceive that medical tuition, when at all successful, is one of the most powerful and permanent means of acquiring, extending, and securing medical practice. The mind of youth is naturally grateful as well as susceptible; he is easily carried away by the lessons of his preceptor—imbibes his doctrines and ideas—and forms a kind of filial affection in return for early instruction. On all subsequent occasions, he not only consults his master in the moment of doubt or difficulty, but recommends him to his patients.

We believe we assert no more than the truth, when we say that the publication of Dr. Armstrong's lectures did not enhance his reputation among the higher orders of the profession, who were best judges of their merits. No man, however superlative his talents, however unlimited his acquirements, need expect to shine in the whole circle of medical science, embraced in a course of lectures. He then loses the advantage of concentrating his powers towards a single subject—and necessarily becomes a compiler — the retailer of what others had said, done, and taught before his time. Dr. Armstrong's lectures, with all the pains which he had taken, were very different compositions from that of his work on typhus. Nevertheless, they extended his name and fame among the great mass of professional and extra-professional society—they increased his practice—and they brought him in a very considerable revenue.

The tide of prosperity had now floated him triumphantly over all the shoals and quicksands on which so many are stranded—and was carrying him steadily and rapidly forward to fortune and independence, with every rational prospect of providing for a large and beloved family. But it was quite evident to all who saw Dr. Armstrong, that he was over-working himself—and that the WEAR and TEAR of his preceptorial and professional avocations was far too great for the constitution to withstand for any length of time. The habit of lecturing, in such a place as the Borough, in Summer as well as Winter—the inhalation of putrid effluvia during the examination of morbid parts—the fatigue and anxiety of an extensive private practice —and above all the great, the dangerous, and, we may add, the unnecessary excitement into which he worked himself during the delivery of almost every lecture, were sufficient to consume the frame of a Hercules—and much more a frame in which already lurked the germ of a fatal malady. Dr. Armstrong never exhibited the marks of a sound, or at all events, of a strong constitution. He was susceptible of colds, and often affected with cough, which was, as is usual in such cases, attributed to mere catarrhal affection of the mucous membrane. During the last 18 months, however, the pulmonary affection assumed a more formidable shape, at least in the eyes of his friends—and during the Summer of the year just ended, he was obliged not only to desist from lecturing, but to retire into the country for change of air. He went first to Dorking, then to Worthing, and afterwards to Dorking again, whence he returned to London about the end of August or beginning of September, apparently improved in health, and to use his own expression, "quite well." But the cough had never entirely ceased, and he was considerably emaciated. He went down in September to the North of England, his native country, and returned in October, very much altered for the worse. The prominent moral character of phthisis shone forth in this talented physician as strongly as in the most unlettered peasant—utter incredulity as to the actual nature of the disease!

Dr. Armstrong would not admit, for a moment, that there was any serious disease of the lungs in his case—and we believe that a gentleman with whom he was closely connected, got into some disgrace by saying that "Dr. Armstrong himself began to admit that he was consumptive." So sanguine was this amiable physician of recovery, that even in the month of November last, he took considerable pains to counteract the above report, and authorised several of his friends to contradict it on his own special authority—declaring that he had never entertained or expressed such an opinion as to his own case.

Whatever danger might have accrued from this confidence of security at an earlier period, before the malady had made progress, and when many of the exciting causes might have been avoided, it was now perhaps a "blindness to the future wisely given" — since phthisis had actually advanced almost to the last stage! Still Dr. A. continued to go out in his carriage, till within ten or fourteen days of his death, which took place, without a struggle, on Saturday night, the 12th December, in the 46th year of his age!

The emaciation was such that no person could have recognized the countenance of the living in the dead body! On examination, an immense excavation was found in the left lung, capable of containing a man's fist, with tubercles and adhesions in the same side. The right lung, especially towards the summit, was studded with tubercles, some of them very hard, others beginning to soften down.

Thus has terminated the short but brilliant career of one of the most talented and amiable physicians of the present century! We, who have been his warmest advocates through life, have freely expressed our opinions respecting his defects, now that the tomb has closed over his mortal remains—while those who denied him any merit while alive, will probably overwhelm his memory with unbounded praise! If it be asked why we did not proclaim our opinions respecting the failings of Dr. Armstrong before his death, we answer—*first*, we did not choose to injure the *living*, supposing that it was in our power so to do—*secondly*, we cannot injure the *dead* by any thing that we now may say—and *thirdly*, we may benefit the *living*, full as much by pointing out the failings that are to be avoided, as the virtues that are to be imitated in a public character.

The short but eventful life of Dr. Armstrong, is capable of furnishing a useful moral lesson to his brethren left behind. We do not believe, indeed, that moral lessons drawn from the dead, make any very lasting impression on the living. The passions of the present soon overcome the precepts of the past, and thus we find man running the same giddy round of ambition, pleasure, avarice, love, and other human impulses, with little or no reference to the dangers into which their predecessors had fallen in the same course. The members of the medical profession, however, are a thinking people, and we believe that the premature death of one of their most distinguished contemporaries, may afford them wholesome food for a moment's reflection.

Dr. Armstrong, in the garb of humility itself, was one of the most ambitious physicians of the age or country in which he lived. By the word "ambitious," we mean to cast no stigma on his character,—quite the contrary. His was that laudable ambition which carries men to the head of their profession, whatever it may be, and without which they never can rise beyond mediocrity. But that very ambition, laudable as it was, destroyed his life! Had he aimed at less, he would have accomplished more. The wear and tear of mind, occasioned by the compilation of his lectures, gave the first shock to his health: the incessant excitement into which he was ever worked by the delivery of those lectures, together with the exertion of voice inseparable from such delivery, brought into activity those dormant germs of phthisis which he carried with him perhaps from his mother's womb. Had this talented physician been contented with the fine and prosperous gale with which he sailed during the first few years of his career in London; had he allowed himself some relaxation, and fresh air every Summer, instead of wearing himself out in the mephitic atmosphere of the Borough, he would, in all human probability, have now been in as good health as when he first approached the metropolis. That he had always tubercles in his lungs there can be little doubt, from the immense number of these bodies found after death, and the state of induration in which many of them existed. That they should have been called into rapid and fatal activity after the age of 44, without strong and unusual exciting causes, is contrary to all human experience and reasoning. These extraordinary causes, we conceive, are clearly referrible to the excessive wear and tear of constitution in the deceased's case, aided by the inordinate excitement and exertion of the respiratory apparatus, resulting from lectures carried on during all seasons, in different localities, and sometimes more than once or twice in the same day! In fine, we have not the slightest doubt that this amiable and highly gifted physician, fell a sacrifice to the attempt at undergoing a greater amount of intellectual and corporeal labour, than any constitution, or, at all events, his constitution could sustain without inducing premature decay ! This may be a lesson to those few of his surviving brethren, on whom public fame, and, what is usually considered their lucky stars, have thrown the weight of extensive practice. Let them remember that the human machine, moral and physical, has its range of action, beyond which it is not quite safe to urge its speed!

"All men think all men mortal but themselves."

Yet a day comes at length, when the finest mind, the firmest nerve, and the toughest muscle, refuse to obey the most urgent call that can be made on them by the thirst of ambition, or that last of passions, the love of gold !

